# Epidemiologia das Doenças Cardiovasculares no Brasil: A Verdade Escondida nos Números

**DOI:** 10.36660/abc.20200793

**Published:** 2020-08-19

**Authors:** Carisi Anne Polanczyk

**Affiliations:** 1 Hospital de Clinicas de Porto Alegre Porto Alegre RS Brasil Hospital de Clinicas de Porto Alegre,Porto Alegre, RS - Brasil; 2 Hospital Moinhos de Vento Porto Alegre RS Brasil Hospital Moinhos de Vento,Porto Alegre, RS – Brasil; 3 Faculdade de Medicina Universidade Federal do Rio Grande do Sul Porto Alegre RS Brasil Faculdade de Medicina da Universidade Federal do Rio Grande do Sul,Porto Alegre, RS – Brasil

**Keywords:** Doenças Cardiovasculares/mortalidade, Confiabilidade de Dados/tendências, Sistemas de Informação em Saúde/tendências, Formulação de Políticas, Epidemiologia

Segundo a Agenda 2030 da Organização Mundial de Saúde para o Desenvolvimento Sustentável, existe um comprometimento dos países-membros para uma redução de 30% na mortalidade prematura por doenças não transmissíveis, particularmente as doenças cardiovasculares (DCV) (cardiopatia isquêmica e acidente vascular cerebral — AVC), câncer, doença respiratória e diabetes.^1^ Essas condições são responsáveis por aproximadamente 41 milhões de mortes por ano, equivalente a 71% das mortes no mundo.^2^ Para direcionar estratégias de enfrentamento dessas doenças, informações provenientes de sistemas confiáveis, transparentes e reprodutíveis são essenciais. A análise de tendências de mortalidade é crucial para o desenvolvimento efetivo de políticas de saúde, seguridade social, investimentos, entre outras.

A iniciativa dos estudos do *Global Burden of Disease (GBD)* vem ao encontro dessa agenda, tendo como objetivo aprimorar o entendimento das doenças através da análise de dados disponíveis sobre a incidência, prevalência e mortalidade de modo consistente, atualizado, global, em âmbito regional e nacional.^3^ Ao longo dos últimos anos, esta proposta metodológica trouxe informações práticas para o enfrentamento das doenças ao redor do mundo, vencendo desafios inerentes à metodologia, particularmente a heterogeneidade dos registros e dados oriundos dos diversos países. ^4 , 5^ Através de dados provenientes de múltiplas fontes (registros de saúde, coortes e ensaios prospectivos, dados administrativos, análise verbal, entre outros) e aplicando modelos estatísticos complexos, a iniciativa tem fornecido dados por sexo, idade e país, de mais de 310 doenças e agravos, com aprimoramento metodológico constante. ^6^

A importância dessas novas métricas são apontadas no artigo de Malta et al.^7^ Os autores compararam séries históricas de mortalidade por DCV entre 2000 e 2017, provenientes de três estimativas: dados do Sistema de Informação sobre Mortalidade (SIM) brutos, corrigidos para causas mal definidas e sub-registros e aquelas aplicadas pelo GBD. Ao longo desse período, em todas as bases, houve uma redução da mortalidade por DCV no Brasil, de 27% no SIM bruto e 28% pelo GBD. Entretanto, subanálises por unidades da federação mostraram como os dados do SIM bruto podem ser equivocados. Segundo registro do SIM, em 12 estados, houve aumento do número de mortes atribuídas às DCV, enquanto pelas estimativas do GBD, em todos estados, houve redução da mortalidade por essas doenças. Fato este relevante para o monitoramento das ações de prevenção e controle pelos gestores e pela sociedade. Entretanto, o ponto a ser destacado é ainda o percentual elevado em alguns estados de preenchimento inadequado de registros de óbitos, causas mal definidas, sendo, em 2017, 42% ainda classificados como códigos errados ( *garbage code* ).

A metodologia empregada pelo GBD visa padronizar internacionalmente as causas de morte, que na sua origem são estabelecidas em um registro único de um médico. Devido à ampla variabilidade nesses aspectos, tratamento com algoritmos e modelagem permitem que uma proporção de causas de morte mal definidas ou classificadas como outras sejam realocadas para causas mais prováveis.^8^ Ponto sensível da metodologia é a inferência para alguns códigos, denominados “ *garbage codes* ”. Alguns são intuitivos, como causas mal definidas ou sintomas; outros são sujeitos a interpretação e arbitrariedade. Por exemplo, insuficiência cardíaca é compreendida como causa intermediária de morte, e as mortes atribuídas a esse código são reclassificadas através de modelo de regressão considerando idade, sexo e localização. A precisão e acurácia desses ajustes a cada realidade é algo a ser explorado. Certamente, a metodologia do GBD nos traz luz para desvendar os casos obscuros de registro. Entretanto, o lado obscuro existe e precisa ser continuamente trabalhado. Para confiarmos mais nessas estimativas, devemos buscar melhor qualidade no registro de base original.^4 , 8^

O Brasil é um país de dimensões continentais, com uma das maiores desigualdades socioeconômicas, uma situação que inevitavelmente está relacionada com maior mortalidade por doenças não transmissíveis, especialmente DCV.^9^ O envelhecimento da população, globalização, urbanização com aumento da obesidade e inatividade física são fatores determinantes desses números. Nas últimas décadas, felizmente muito foi alcançado e reduzimos de modo expressivo a mortalidade por essas condições em todos os estados. Entretanto, sabemos que há muito a ser feito; existem desigualdades imensas nesses números, parte expressiva relacionada a fatores como baixo nível de estrutura e recursos na saúde, baixo nível socioeconômico e cultural da população. O mais preocupante é saber que em condições de recursos escassos, os custos com tratamento das DCV acabam drenando mais ainda os recursos existentes, gerando um círculo vicioso de mais pobreza e atraso para o crescimento.

Nosso desafio é como levar esses dados para além da academia e dos cientistas. Como fazer que as estimativas de prevalência, incidência e fatores de risco das doenças cardiovasculares sejam empregadas por gestores e políticos para tomada de decisão?^4 , 7^ O primeiro passo, dado por Malta et al.,^7^ é transformar registros existentes em informações relevantes e válidas que possam nortear ações objetivas de controle das DCV. A aceitação de que registros brutos locais são insuficientes para este propósito é extremamente relevante. Por outro lado, é fundamental que estes dados sejam empregados por gestores, tomadores de decisão, organismos não governamentais e certamente pela comunidade médica, para entender melhor as doenças da nossa população e reavaliar esforços, identificar ações prioritárias para combate e melhorias para a saúde cardiovascular no Brasil.
