# Supplementary material for: Epidemiologia das Doenças Cardiovasculares no Brasil: A Verdade Escondida nos Números
Source: Arq Bras Cardiol. 2020 Aug 19;115(2):161–2. [Article in Portuguese] doi: 10.36660/abc.20200793 (PMC8384285; doi:10.36660/abc.20200793)
Supplement: Translation

# Translation: Epidemiology of Cardiovascular Diseases in Brazil: The Truth Hidden in the Numbers

**Authors:** Carisi Anne Polanczyk

**Affiliations:** 1 Brazil Hospital de Clinicas de Porto Alegre, Porto Alegre, RS - Brazil; 2 Brazil Hospital Moinhos de Vento, Porto Alegre, RS – Brazil; 3 Brazil Faculdade de Medicina da Universidade Federal do Rio Grande do Sul, Porto Alegre, RS – Brazil

**Keywords:** Cardiovascular Diseases/mortality, Data Accuracy/trends, Health Information System/trends, Policy Making, Epidemiology

According to the 2030 Agenda of the World Health Organization for Sustainable Development, member states are committed to a 30% reduction in premature mortality from non-communicable diseases, particularly cardiovascular diseases (CVD) (ischemic cardiomyopathy and stroke), cancer, respiratory diseases and diabetes.^1^ These conditions account for about 41 million deaths per year, i.e., 71% of deaths worldwide.^2^ To direct strategies for to prevent and treat these with these diseases, information from reliable, transparent and reproducible systems is essential. The analysis of mortality trends is key for the effective development of health, social security, investment and other policies.

The Global Burden of Disease (GBD) studies initiative is in line with this agenda, as it aims to improve understanding of diseases through the analysis of data on incidence, prevalence and mortality in a consistent, updated and global manner, both at a regional level and at a national level.^3^ Over the past few years, this methodological proposal has brought practical information for reducing these diseases around the world, overcoming challenges regarding methodology, particularly the heterogeneity of records and data from different countries.^4 , 5^ Through data from multiple sources (health records, cohorts and prospective trials, administrative data, verbal analysis and others) and applying complex statistical models, the initiative has provided data by sex, age and country, to more than 310 diseases and conditions, with continuous methodological improvement.^6^

The importance of these new metrics is pointed out in the article by Malta et al.^7^ The authors compared historical series of CVD mortality between 2000 and 2017, based on three estimates: gross data from the Mortality Information System (SIM), corrected for ill-defined causes and underreporting, and those applied by the GBD. Over this period, on all databases, there was a reduction in mortality from CVD mortality in Brazil, by 27% in the gross SIM and 28% by the GBD. However, sub-analyses by state have shown how inaccurate the SIM data can be. According to the SIM records, in 12 states, there was an increase in the number of deaths from CVD, while according to the GBD estimates, in all states, there was a reduction in mortality from these diseases. This fact is relevant for the monitoring of prevention and control actions by managers and society. However, a fact to be pointed out is the high percentage, in some states, of death records and ill-defined causes improperly filled out, and, in 2017, 42% are still classified as garbage codes.

The methodology used by the GBD aims to internationally standardize the causes of death, which, in their origin, are established in a single medical record. Due to the wide variability in these aspects, treatment with algorithms and modeling allows a proportion of ill-defined deaths or deaths classified as other causes to be reallocated to more probable causes.^8^ A sensitive point of the methodology is the inference for some codes, called “garbage codes”. Some are intuitive, like ill-defined causes or symptoms; others are subject to interpretation and discretion. For example, heart failure is understood as an intermediate cause of death, and deaths attributed to this code are reclassified using a regression model considering age, sex and location. The precision and accuracy of these adjustments to each reality is something to be explored. Certainly, the GBD methodology brings us light to unveil the obscure cases of records. However, the dark side exists and needs to be worked on continuously. To rely more on these estimates, we must seek better quality in the original baseline record.^4 , 8^

Brazil is a country of continental dimensions, with one of the greatest socioeconomic inequalities, a situation that is inevitably related to higher mortality from non-communicable diseases, especially CVD.^9^ Population aging, globalization, urbanization with increased obesity and physical inactivity levels are determining factors in these numbers. Fortunately, much has been achieved in the past few decades and we have significantly reduced mortality from these conditions in all states. However, we know that there is much to be done; there are huge inequalities in these numbers, mostly related to factors such as low level of structure and resources in health, and low socioeconomic and cultural level of the population. The most worrying thing is to know that in conditions of scarce resources, the costs of treating CVD end up depleting the existing resources even more, generating a vicious circle of more poverty and delayed growth.

Our challenge is taking this data beyond academia and scientists. How can the estimates of prevalence, incidence and risk factors for cardiovascular diseases be used by managers and politicians in their decision-making efforts?^4 , 7^ The first step, taken by Malta et al.,^7^ is to transform existing records into relevant and valid information that can guide objective actions to control CVD. Accepting that local gross records are insufficient for this purpose is extremely relevant. On the other hand, it is essential that these data be used by managers, decision makers, non-governmental organizations and certainly by the medical community, to better understand the diseases of our population and to reassess efforts, identify priority actions to combat and improve cardiovascular health in Brazil.
